# Response to the paper “unraveling functional Neurology: an overview of all published documents by FR Carrick, including a critical review of research articles on its effect or benefit.” by Marine Demortier and Charlotte Leboeuf-Yde

**DOI:** 10.1186/s12998-020-0298-z

**Published:** 2020-01-28

**Authors:** Frederick Robert Carrick

**Affiliations:** 1 Professor of Neurology, University of Central Florida College of Medicine, Orlando, FL USA; 2 Adjunct Professor, MGH Institute for Health Professions, Boston, MA USA; 3 Senior Research Fellow Centre for Mental Health Research (CMHR-CU) in association with University of Cambridge, Cambridge, UK

A functional neurologic disorder is a prevalent and disabling condition at the intersection of neurology and psychiatry [1] with a current PubMed search of the term Functional Neurology (FN) listing 44,939 articles [2], 2618 Systematic Reviews [3] and 191 Meta-Analysis [4]. A PubMed search of my name and FN finds one publication addressing persistent vegetative state as a pejorative term [5] and another addressing the metrological performance of instruments used in clinical evaluation of balance [6]. However, Demortier and Leboeuf-Yde reviewed 121 of my publications through October, 2018 to measure the effect/benefit of treatment/intervention using a FN approach when I have not written anything on the subject.

Functional neurology represents a paradigm of healthcare that utilizes an evidence-based approach to quantify human performance and function. It does not represent a theory or hypotheses or any diagnostic or treatment modalities [7]. Demortier and Leboeuf-Yde state that the scientific basis of FN was my 1997 publication describing changes in the size of the blind spot by somesthetic stimulation [8] rather than my earlier publications addressing lumbar [9] and cervical radiculopathy [10]. However none of these works represent the scientific basis of FN. I did not suggest a clinical application to my observations of changes in the functional size of the physiological blind spot but others have recently demonstrated that it can also be shrunk through exercises with significant clinical applications [11].

There is an abundance of evidence-based literature reporting diagnostic and treatment modalities under a FN paradigm. Functional connectivity (FC) [2, 12–14], functional neuroanatomy of sequence learning [15], functional neural substrates of reward processing and inhibitory control [16], functional brain network connectivity [17], functional neurological characterization of disease [18], functional exercise capacity [19], utilization of functional magnetic resonance imaging (fMRI) scanning of cognitive tasks [20] are all components of a FN paradigm and the list goes on and on. While physical exercise is an evidence-based application within the paradigm of FN, it would not and should not be classified as a FN treatment. A search of the literature to discover an increase in muscle strength by a FN treatment would come up short and so it should. Gait and balance impairments are FN paradigm examples of treatable targets that can manage reduced functional mobility [21, 22] but they are not FN treatments.

In an attempt to find evidence for FN, the authors have elevated select empiricism over other forms of knowledge important to clinicians, patients and society. This is problematic in this case as their conclusions are made based upon a review of my published work that does not reflect the topic of their interest.

## Data Availability

None.
